# Post-policy evaluation lags of tobacco and nicotine product policies: a systematic review from the United States, Canada, Australia, and the United Kingdom

**DOI:** 10.3389/fpubh.2026.1895110

**Published:** 2026-09-29

**Authors:** Huma Aziz, Andrea M. Hayes, Omotayo Francis Fagbule, Ene Ikpebe, Bukola Peters

**Affiliations:** 1 Department of Public health, Purdue University, West Lafayette, IN, United States; 2 School of Public Health, University of Nevada Reno, Reno, NV, United States; 3 Askew School of Public Administration and Policy, Florida State University, Tallahassee, FL, United States

**Keywords:** cigarette use, e-cigarette use, evaluation lag, federal policy, nicotine-control, tobacco-control

## Abstract

**Objective:**

This systematic review examines the time interval between policy implementation and evaluation of behavioral results in tobacco control studies (evaluation lag) as well as reported estimates. Results can help policymakers avoid premature conclusions and make more informed decisions about policy effectiveness.

**Methods:**

We reviewed studies from 2010 onward evaluating the effects of policies implemented at national-level or across majority provinces on behavioral outcomes, including tobacco use, cessation attempts, and secondhand smoke exposure, in the United States, Canada, Australia, and the United Kingdom. Eligible studies were published in the English language. The search databases included PubMed, Web of Science, Embase, Google Scholar, and government websites. Two reviewers independently screened studies according to predefined eligibility criteria and extracted data. A third reviewer resolved conflicts. Study quality was assessed via the JBI Critical Appraisal Checklist.

**Results:**

A total of 21 studies met the inclusion criteria. Studies evaluated policies across various time points ranging from 6 months to 1 year (*n* = 5), one to 2 years (*n* = 12), more than 2 years (*n* = 5) and multiple time points (*n* = 2). The policies evaluated included smoke-free (n = 4, 19.1%), retail-related (*n* = 14, 66.6%) and nicotine-related (*n* = 3, 14.3%). In the risk of bias assessment, 19 studies had low risk (90.5%) and two studies had uncertain risk (9.5%). Most of the smoke-free policies were evaluated earlier (within 6 months to 1-year). Most of the retail and nicotine- related policies were evaluated 1 year and above after implementation. Most of the studies showed positive results after policy implementation.

**Conclusion:**

Policy evaluation lags vary by policy type with smoke-free policies more frequently evaluated earlier than other tobacco and nicotine control policies. These findings highlight the importance of identifying optimal evaluation periods according to policy type.

**Clinical trial registration:**

https://www.crd.york.ac.uk/PROSPERO/view/CRD420251175482, identifier PROSPERO (CRD420251175482).

## Introduction

Tobacco and nicotine product policies are population-level strategies implemented to decrease their use and associated harmful effects. These policies aim to prevent initiation, promote cessation, and reduce exposure to secondhand smoke. Smoke-free laws, for example, restrict the use of tobacco or nicotine products in public spaces, whereas retail-related policies control the availability, marketing, pricing, and product characteristics of tobacco through measures such as advertisement bans, setting a minimum legal sales age to purchase products, health warning labels, plain product packaging, increased taxes, and flavor bans (1).

Public health policy evaluation studies often focus on whether policies lead to changes in health or behavior, even though these outcomes usually occur after earlier changes in systems or environments, such as access, pricing, or exposure. While some studies observed changes in outcomes post-policy implementation, there is no consistent evidence on the timing of observed effects across different types of tobacco and nicotine policy evaluations. Different studies assess policy outcomes at different times, ranging from 6 months after the policy passed to as late as 8 years after policy implementation (2–6). However, due to the substantial variability in when outcomes are assessed following implementation complicates interpretation of policy impacts and limit comparability across studies. Such information on the timing from implementation to evaluation can be gathered from existing studies (7–9). Studies have assessed several policies, including smoke-free laws, flavor bans, and sale to minor laws, at different times with varying results—no changes, positive (behavioral improvement), negative (worsening of behavior), or mixed. While many studies focus on whether tobacco control policies are associated with changes in outcomes, fewer studies explicitly examine the timing of these evaluation studies following policy implementation. To examine post-policy periods and how they vary by policies, we asked several questions: (1) when do researchers typically measure policy outcomes post-implementation in the current literature, and (2) do the evaluation periods differ depending on type of policy? We also sought to synthesize findings on general impacts, i.e., positive, negative or no change in the behavioral outcomes. Answers to these questions will help researchers identify optimal evaluation periods and will significantly improve policy-making strategies by guiding policymakers in effective policy design, adoption, implementation, or revision.

To answer these questions and recognize that timely evaluation of tobacco and nicotine control policies can lead to substantial benefits at the population level, we conducted a systematic review of policy evaluation studies from January 1, 2010, to May 27, 2025, specifically examining the timing of these post-policy assessments and their reported outcomes. To keep the study focused, we restricted the review to tobacco and nicotine product control policies implemented at the national-level or across majority provinces in the United States, United Kingdom, Canada, and Australia. These high-income countries have implemented a broad range of tobacco and nicotine control measures and have an extensive evaluation literature, providing useful insight into the timing from policy implementation to evaluation. In this review, we focus on the time interval between policy implementation and when outcomes were evaluated in published studies. Although this measure does not necessarily capture the exact point when change first occurs, it provides insight into how long after adoption researchers typically detect and report measurable impacts of tobacco and nicotine control policies.

## Methods

A systematic review was conducted to answer our research questions. We followed the methodology outlined in the review protocol registered with PROSPERO (CRD42023485394). We used the Population, Intervention, and Outcome (PIO) framework to develop the search strategy (10, 11). Our population of interest was studies evaluating tobacco and nicotine control policies implemented in the United States, Canada, Australia, and the United Kingdom. The intervention of interest was tobacco and nicotine control policies. The outcome of interest was the reported timing between policy implementation and study evaluation in outcomes such as tobacco use or prevalence, secondhand smoke exposure, quit attempts, and cessation. Details of the search strategy, inclusion criteria, data sources, screening process, and data extraction are given below.

### Search strategy

We conducted an electronic search for eligible studies published from January 1, 2010, to May 27, 2025, across multiple databases, including Web of Science, Scopus, Embase, and PubMed. Google Scholar and government websites were searched for any additional literature and government documents not indexed in the above mentioned databases. An initial search strategy was developed and peer-reviewed by a librarian as best practice (12), after which the search terms were refined and updated prior to the final search. The main keywords used included: “federal or national or country or nation”, “public policy or legislation or policy or restrictions or prohibitions or ban or statute or law or regulation or tobacco control”, “tobacco or smoking or nicotine or e-cigarettes or vape or cigar”, and “United States or United Kingdom or Canada or Australia”. The complete search strategy used for all databases is given in the online supplemental material (Supplementary Table 1 and Supplementary Text).

The results from individual databases were uploaded to Covidence, a web-based systematic review platform, to enhance collaboration and streamline the screening process. A two-step screening process (title/abstract and full-text screening) was independently conducted by two reviewers to reduce selection bias and ensure that only studies meeting the specified criteria were included in the final analysis. Conflicts were resolved either through discussion between reviewers or adjudication by a third reviewer.

### Inclusion criteria

We included studies that examined policies implemented from January 2010 until May 2025 at the national level and across the majority of provinces or states and were evaluated using nationally representative samples. We limited our review to four high incomes countries (HIC), the United States, the United Kingdom, Canada, and Australia. Our selection was based on their well-established tobacco-control regulatory framework, availability of well-documented policy implementation dates and extensive literature on policy evaluation published in English language, essential for analyzing policy-to-evaluation lag times for this review. We acknowledge the exclusion of other relevant countries with active tobacco control framework and extensive evaluation studies, such as New Zealand and European countries. New Zealand was excluded because its major policies were implemented before 2010, outside the review period, and its efforts focused on education and cessation rather than new policies. European countries were excluded due to differences in policy governance (where EU Directives roll into national laws at very staggered rates) which make policy implementation lag difficult to follow. Also, analyzing these countries would require evaluating non-English literature, raising the risk of translation gaps in our lag-time data Selection of these four HIC ensures comparability and relevance to the tobacco and nicotine control literature of the past decade and limits the heterogeneity of the review.

We included articles or original study articles published in the English language as full papers, including quasi-experimental study designs, pre- and post- study designs, cross-sectional study designs, cohort studies and interrupted time series (randomized controlled trials, simulation studies, and systematic reviews were excluded). We included articles that focused on short-term behavioral outcomes such as tobacco use or prevalence, secondhand smoke exposure, quit attempts and cessation. We included articles that clearly stated the implementation dates of the policies they evaluated. We excluded studies evaluating guidelines or recommendations and studies with no clear policy implementation dates.

### Measures

The outcome of interest was the reported timing between policy implementation and study evaluation in the short-term public health outcomes mentioned in the previous section. We included the term ‘implementation date’ as the date the policy came into effect, as stated in the studies we reviewed. ‘Post policy evaluation lag’ (PPEL) was defined as the time taken between the implementation date and the evaluation of outcome of interest.

We categorized policies as smoke-free, retail-related, nicotine-related, and multiple policies. The ‘Smoke-free’ category includes policies implemented to ban smoking in public or private places. The ‘Retail-related’ category includes policies such as flavor bans on tobacco products (e.g., cigarettes and cigars), product packaging such as standardized and plain packaging, warning labels, point of sale bans such as on tobacco displays, sales, and licensure. The ‘Nicotine-related’ category included policies related to e-cigarette packaging, access, advertisement, nicotine strength and flavor bans.

### Implementation dates

While there were several policy evaluation studies, as mentioned earlier, for consistency, we only included articles that clearly mentioned the implementation dates of the policies being evaluated. For policies that had a phase-in period from the effective date, we used the last date of the phase-in period as the implementation date. For policies implemented at provincial/state levels, we have included provinces/states that had implemented policy in 2010 or onwards (see Supplementary Table 2). For studies that examined the policy at multiple time points and contributed to more than one PPEL category, we reported the results for all the time points included in the study (see Table 1).

**Table 1:** Summary statistics of included studies (*n* = 21).

Policy tool	Policy Evaluation Timing			
	Within 6 months to 1 year (*n* = 05)	1 year to 2 years (*n* = 12)	Over 2 years (*n* = 05)	Multiple time points (*n* = 02)
Retail-related policy (e.g., Graphic health warnings, flavored tobacco sale restriction, minimum legal sales age, standardized packaging, tax etc.)	East (15) (+) Opazo Breton (16) (+)	Durkin (29) (+) Thrasher (20) (+) Carpenter (21) (+) Chung Hall (22) (+) (−) Chung Hall (30) (+) (−) Gray Lit 1 (27) (+) Gray Lit 2 (28) (−) Agaku (23) [Oregon, Maine, and Massachusetts] (+) Havard (24) (+)	Buss (32) (no effect) Kuipers (33) (+) Underwood (34) (+) Agaku [California and Hawaii] (23)(+)	
Smoke-free policy	Tweed (17) (+) Thorpe (18) (no effect) Elton-Marshall [Manitoba] (19) (+)	Hunt (25) (+) Elton-Marshall [Newfoundland/Labrador] (19) (+)		Within 6 months to 1 year: Elton-Marshall [Saskatchewan] (19)(+) 1 year to 2 years: Elton-Marshall [Saskatchewan] (19)(no effect)
Nicotine-related policy (e.g., nicotine strength, sales restrictions etc.)		Moore (26) (+) (−)	Mendelsohn (31) (+)	Within 6 months to 1 year: Hammond (35) (−) 1 year to 2 years: Hammond (35) (−)

Studies are not mutually exclusive in each category. Studies evaluating policy at multiple time points post-implementation are summarized separately for the specific geographic locations for the easy of understanding.(+) shows a reduction in tobacco/nicotine product use, (−) shows an increase in tobacco/nicotine product use and (no effect) shows no change in tobacco/nicotine product use.

### Quality assessment

A systematic quality assessment of the included studies was conducted to evaluate the risk of bias via the JBI Checklist (13). Each study was assessed for internal and statistical validity using four predefined criteria for each (see Supplementary Table 3). Studies that met at least six out of the eight criteria were categorized as having a low risk of bias. Those that met no more than four criteria were deemed to have an unclear risk of bias, whereas studies that met two or fewer criteria were classified as having a high risk of bias.

### Data extraction

A data extraction template available from Covidence was used to extract the study characteristics and was pilot tested on the first five studies included in the review to make changes that allowed the addition of relevant details from each study. Two reviewers independently extracted the data. A consensus was reached after discussion between the two reviewers. We extracted the following information: study identification (authors’ details, study setting, start and end date etc.), methods used (study design, aim, unit of analysis, measures), intervention (policy evaluated, implementation dates as stated within the study, evaluation date which was when the data were collected [not when the researchers conducted the analyses]), and study outcomes (prevalence estimates pre- and post-policy implementation and estimates from the fully adjusted models for multivariable models).

Given the heterogeneity of the study designs and tobacco control policies, we conducted a systematic narrative synthesis to answer our research questions, adhering to the PRISMA 2020 guidelines (14). The synthesis process was carried out collaboratively by three reviewers to enhance reliability. PRISMA checklist is provided in the supplementary document.

## Results

The results of the search selection are shown in the PRISMA flowchart in Figure 1. During the screening process, 164 studies were deemed eligible, but only 21 studies were included in the final sample for data extraction. The subsequent sections focus on the characteristics, the quality assessment, and the findings from the included studies.

**Figure 1:**
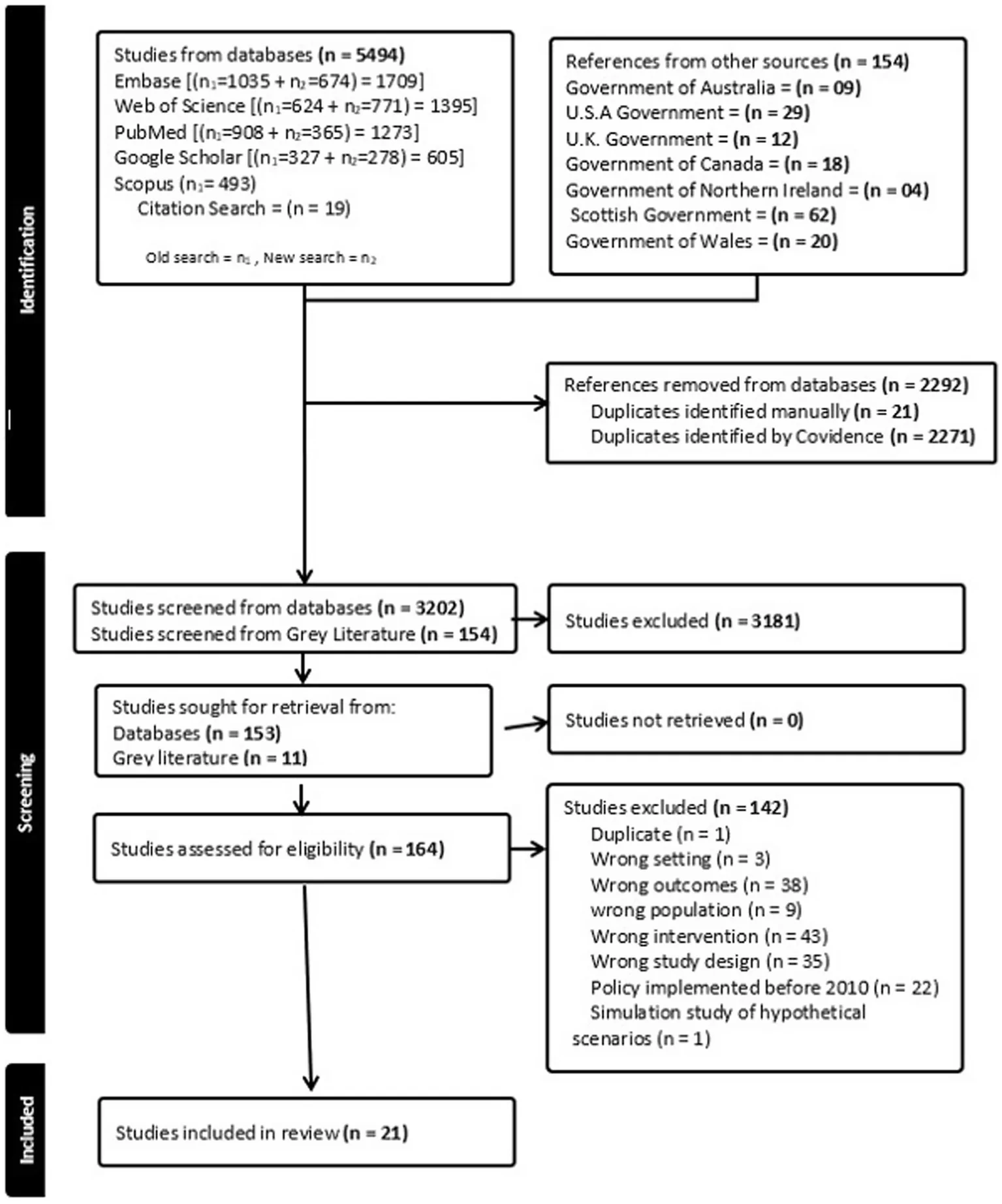
PRISMA flow diagram of study selection process.

### Characteristics of the studies

Some studies in our sample contributed to multiple PPEL categories and are thus not mutually exclusive. Of the 21 studies included in the final sample (Figure 2; Supplemental Table 2), five measured the primary outcome within 6 months to 1 year post-implementation (15–19), 12 measured outcomes 1 to 2 years post-implementation (19–30), five measured outcomes two or more years post-implementation (23, 31–34) and two measured outcomes at more than one post-implementation time periods (19, 35).

**Figure 2:**
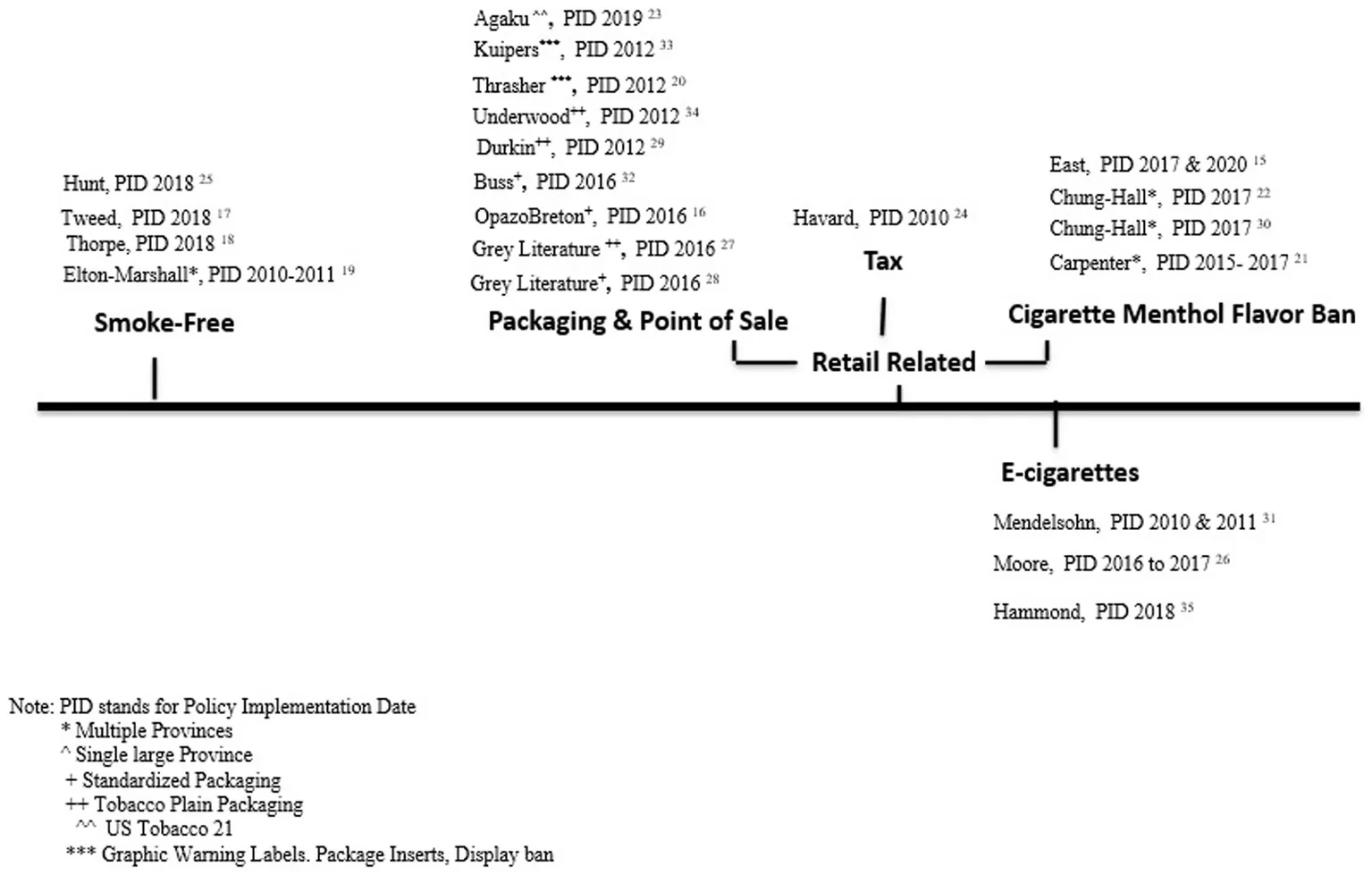
Evaluation studies, tobacco policy types, and implementation dates (*n* = 21).

Among the included studies, three used quasi-experimental design to evaluate policy, six used cohort study design, two used case–control design, two were governmental reports, two used both quantitative and qualitative methods, and six used cross-sectional data to evaluate policy. Of the 21 studies, two (9.5%) evaluated policies implemented within the United States and were focused on smoke-free and sales related policy (18, 23), four (19%) evaluated policies implemented in Australia (24, 29, 31, 34) with primary focus on packaging along with nicotine and tax related policies, six (28.6%) evaluated policies implemented in Canada with more than half focused on menthol cigarette ban (19–22, 30, 35), eight (38.1%) evaluated policies implemented in the United Kingdom (16, 17, 25–28, 32, 33) with more than half focused on standardized packaging and one study (4.7%) evaluated menthol flavor ban in Canada, England and United States (15) (see Table 2).

**Table 2:** Sample characteristics of included studies (*n* = 21).

Study	Country	Sample size (*n*)	Study design	Policy	Outcomes
Elton-Marshall (19)	Canada	91,800 2004 (*n* = 16,644), 2006 (*n* = 18,257), 2008 (*n* = 19,177), 2010 (*n* = 19,553), and 2012 (*n* = 18,169)	Quasi-experimental	Smoke-free	Exposure to smoking in cars
Thorpe (18)	United States	21 buildings. 1,030 household enrolled in longitudinal air monitoring	Quasi-experimental	Smoke-free	Change in airborne nicotine concentration. Change in particulate matter <2.5 μm
Tweed (17)	Scotland	44,660 individuals incarcerated in enclosed estate. 44,775 individuals from all prisons	Quasi-experimental	Smoke-free	Change in dispensing rates of medications for nicotine dependence and smoking related conditions
Hunt (25)	Scotland	People in custody included in quantitative analysis. Phase 1; *n* = 2,512 Phase 2; *n* = 1,494 Phase 3; *n* = 757	Mixed-methods	Smoke-free	Change in particulate matter <2.5 μm in diameter. Change in airborne nicotine and smoking beliefs
Carpenter (21)	Canada	Youth = 180,000 Adult = 193,000	Cohort	Menthol flavor ban	Ever use and past 30 days use of cigarettes, flavored cigarettes and e-cigarettes. Smoking quit attempt
Chung-Hall (22)	Canada	1,236	Cohort	Menthol flavor ban	Change in smoking status. Menthol purchase. Cessation behaviors
Chung-Hall (30)	Canada	1,236	Cohort	Menthol flavor ban	Brand and flavor validation. Type of flavor currently used
East (15)	Canada, England, United States	7,067	Cross-sectional	Menthol flavor ban	Past 30-day use of menthol or capsule cigarette
Opazo Breton (16)	United Kingdom	278,219	Cross-sectional	SPoT	Current smoking status
Buss (32)	United Kingdom	257,929	Cross-sectional	SPoT	mean daily roll your own cigarette consumption
Review of Tobacco Legislations (Grey Literature) (27)	United Kingdom	Not provided	Report	SPoT	Smoking prevalence, intention to quit, attempts to quit smoking, cost and affordability of tobacco
Review of Tobacco Legislations (Grey Literature) (28)	United Kingdom	The only data collection was for interviews with 20 sampled retailers	Report	TRPR	Youth and adult cigarette and e-cigarette prevalence, intention to quit and quit behavior
Durkin (29)	Australia	5,441 smokers	Cross-sectional	Tobacco plain packaging	Quit attempts
Underwood (34)	Australia	Not provided. Used nationally indicative time-series data	Case–Control	Tobacco plain packaging	Household tobacco consumption intensity, quantity consumed per week, smoking prevalence
Thrasher (20)	Canada	4,805	Cohort	Health warning inserts	Quit attempts. Reading health warning inserts
Kuipers (33)	England	129,957	Cohort	Point of sale display ban	Current cigarette smoking
Agaku (23)	United States	10,146	Cross-sectional	Tobacco 21	Tobacco consumption, attitudes and perceptions about tobacco access among youth
Mendelsohn (31)	Australia compared to New Zealand	About 13,000–20,000 in each country per wave. 20,000 youth in Australia	Case–control	Nicotine e-liquid regulation	Daily smoking and vaping prevalence
Moore (26)	England, Wales, Scotland	51,056	Mixed methods	TPD regulation	ever and current use of e-cigarettes
Hammond (35)	Canada	12,004 2017 (*n* = 4,024), 2018 (*n* = 3,845) and 2019 (*n* = 4,135),	Cross-sectional	TPVA	Vaping and smoking behavior. Marketing exposure
Havard (24)	Australia	800,619 pregnancies	Cohort	Excise and customs duty increase	Smoking during pregnancy

SPoT, Standardized Packaging of Tobacco Products; TRPR, Tobacco and Related Products Regulation; TPD, Tobacco Products Directive; TPVA, Tobacco and Vaping Product Act.

Evaluation timing varied across policy categories. Studies assessing outcomes within six months to one-year post-implementation were primarily concentrated on smoke-free policies, with fewer evaluations of retail-related policies such as menthol bans and standardized packaging. One study was conducted in United States, two were conducted in Canada, two were conducted in the United Kingdom and one compared policy between Canada, England and the United Kingdom (15–19). Evaluations conducted one to two years after implementation were dominated by retail and product-related policies, including packaging regulations, flavor bans, minimum legal sales age laws, taxation measures, and graphic health warnings. Five studies in this category were conducted in Canada, four were conducted in the United Kingdom, two in Australia and one in United States (20–24, 27–30). During this period, relatively few studies examined smoke-free policies (19, 25) or nicotine-related regulations, with only one study evaluating nicotine-strength regulations under the Tobacco Products Directive in the United Kingdom (26).

Studies evaluating outcomes two or more years post-implementation largely focused on retail-related policies, particularly standardized packaging, minimum legal sales age and graphic health warnings (23, 32–34), while long-term evaluations of vaping regulations (31) and smoke-free policies remained limited. Two of these studies were conducted in the United Kingdom, two in Australia and one in United States. A small number of studies conducted in Canada evaluated policies spanning multiple domains, including the Canadian Tobacco and Vaping Products Act and provincial smoke-free regulations in Canada (19, 35).

Overall, smoke-free policies were more commonly evaluated within shorter time frames (within 6 months to 1 year) and some after two or more years post-policy, whereas retail-related policy studies tended to use longer evaluation lags, ranging from one to several years post-implementation.

### Quality assessment

Out of 21 studies, 19 had a low risk of bias, two had an ‘unclear’ risk of bias and none had a high risk of bias. Table 3 shows the studies included in each category. Details on unclear risk of bias in internal validity and statistical conclusions for the grey literature included in the review are provided in the supplementary material (see Supplementary Table 4).

**Table 3:** Risk-of-bias categorization of final sample (*n* = 21).

Risk-of-bias category	Included studies
Low risk of bias (*n* = 19)	Elton-Marshall (19); Thorpe (18); Tweed (17); Hunt (25); Carpenter (21); Chung-Hall (22); Chung-Hall (30); East (15); Opazo Breton (16); Buss (32); Durkin (29); Underwood (34); Thrasher (20); Kuipers (33); Moore (26); Hammond (35); Havard (24); Mendelsohn (31); Agaku (23).
Unclear risk of bias (*n* = 2)	Gray literature (The Standardised Packaging of Tobacco Products Regulations) (27), Gray literature (The Tobacco and Related Products Regulations) (28)
High risk of bias (*n* = 0)	None

### Findings

Table 1 groups all the studies included in the final sample according to when researchers evaluated the policy and summarized the positive, negative, or no effect of policy measured within each PPEL or across multiple PPEL. Overall, majority of studies within each PPEL reported positive results. However, findings from the two grey literature documents with unclear risk of bias should be interpreted with caution as these comprise of data collected from stakeholders through public consultation as well as a review of existing evidence.

Most of the studies evaluating policies within 6 months to 1 year, reported positive results and one reported no significant change in outcome. These included reduced odds of being a smoker after standardized packaging (16), decline in past 30-day menthol use following a menthol ban (15), decrease in exposure to smoking in cars among youth in Manitoba and Saskatchewan (19) and increased dispensing of nicotine dependence treatments and reduced dispensing for smoking-related illnesses following a national smoke-free prison policy (17). In contrast, one study found no significant changes in indoor air nicotine concentrations following the United States federal smoke-free housing policy (18) and one study found a negative change in outcome, reporting increased vaping prevalence following the implementation of the Canadian TVPA (35).

Similarly, several of the studies evaluating policies one to 2 years post-implementation, also reported positive results, and a few mixed or negative results. These included reductions in menthol cigarette use (21), increased quit attempts associated with health warning labels (20, 29), lower cigarette use among young adults following Tobacco 21 policies in Oregon, Maine, and Massachusetts (23), reduced exposure to secondhand smoke among prison staff (25), decrease in exposure to smoking in cars among youth in Newfoundland/Labrador whereas increase in exposure among youth in Saskatchewan (19), decline in smoking among pregnant women after increase in tobacco tax (24) and declines in smoking prevalence following comprehensive EU regulations (28). On the other and, two studies reported negative results such as increased vaping prevalence following the implementation of the Canadian TVPA (35), particularly in provinces with weaker marketing restrictions and increased e-cigarette use following the United Kingdom tobacco product standardized packaging law (27). Three studies reported mixed results. One study showed an increased youth e-cigarette use in England but not Wales (26). Other two studies showed evidence of both quitting and product substitution following menthol bans (22, 30).

Most studies evaluating outcomes two or more years post-implementation also reported positive results, but two studies reported mixed and negative results. These included decrease in smoking prevalence following point-of-sale display bans (33) vaping regulatory policies (31) and Tobacco 21 in California and Hawaii (23). One study (34) reported reduced household tobacco consumption intensity following Australia’s plain packaging law, although no significant change in smoking prevalence relative to New Zealand. One study found no significant change in outcomes following standardized packaging (32).

Overall, published evaluations of smoke-free policies were more commonly conducted within shorter post-implementation periods, whereas evaluations of retail-related and multi-policy interventions tended to occur after longer intervals following implementation.

## Discussion

This review synthesized evidence from 21 studies to examine the variation in tobacco and nicotine control PPEL across the United States, Canada, Australia, and the United Kingdom. The results showed that most policies were evaluated one year or more post-implementation. Within one to two years follow up period most of the studies evaluated retail-related policies such as flavor ban, and standardized packaging laws. Studies with longer follow-up periods of two or more years also mostly focused on retail-related policy measures, including graphic health warnings, standardized packaging, and point-of-sale restrictions. Most of the nicotine-related policies were evaluated one year or more after implementation. On the other hand, studies focused on detecting impacts within the 6 months to one-year post-policy implementation mostly examined smoke-free policies.

An important consideration when interpreting PPEL is the distinction between policy implementation, enforcement, and compliance (36, 37). In many tobacco and nicotine control contexts, particularly for retail-related policies such as flavor bans, point-of-sale restrictions, and e-cigarette regulations, the effective date of a policy does not necessarily coincide with full enforcement or widespread compliance. Policies may include phase-in periods, sell-through allowances for existing products, delayed inspection or penalty schedules, or gradual enforcement ramp-up, all of which can delay observable changes in outcomes. As a result, it is important to acknowledge that longer evaluation lags observed for retail and nicotine focused policies may reflect implementation and enforcement dynamics. Recognizing these distinctions is essential for interpreting evaluation timing and for designing future studies that align outcome measurement with reasonable implementation timelines.

Less than a third of the studies evaluated smoke-free laws, more than half within the first year and the rest after a year or at multiple time points after the policy was implemented. A majority reported positive outcomes with the smoke-free policy. For instance, smoke-free laws were associated with reductions in cigarette consumption and smoking prevalence, consistent with prior evidence of their immediate impact on exposure and behavior change (38–41). Those that evaluated after a year also observed lower rates of secondhand smoke exposure (19, 25). Only one study (18) found no change in indoor air quality following a smoke-free housing policy, which might reflect non-compliance and enforcement challenges as described in previous studies (42, 43). Our study contributes to existing evidence by showing that most of the smoke-free policies evaluated as early as 6 months to 1 year after implementation have shown significant benefits in reducing tobacco use. However, in some cases (18), more time might be needed to observe benefits associated with smoke-free policies.

About half of the studies evaluated retail-related policies mostly after one or two years of the policy being in place. They assessed policies mostly targeted at retail stores, e.g., flavor bans, standardized packaging and graphic health warnings, and point-of-sale display bans. Over 60% of the studies observed positive outcomes such as decreased use of menthol flavored cigarettes following a menthol ban, or increased number of quit attempts associated with standardized packaging and graphic health warnings. These results are consistent with previous evidence that detailed the effectiveness of flavor bans (44–47), graphic health warnings and standardized packaging (48–50). Our study contributes to the existing evidence by showing that most of these policies were assessed and reported one to two years post-policy.

On the other hand, nicotine-related policies were evaluated across all three PPELs. A greater proportion of these policies reported negative or unintended outcomes, particularly increases in e-cigarette use following vaping restrictions within similar evaluation periods. These findings are consistent with existing evidence showing that the impacts of e-cigarette regulations are heterogeneous and often context-dependent (51–53). Such mixed findings may reflect substitution effects (54), the rapidly evolving tobacco and nicotine product market (55, 56), and inconsistencies in regulatory scope and enforcement across jurisdictions, which together complicate the interpretation of policy impacts over shorter and intermediate time horizons. Unlike smoke-free and many retail-focused tobacco policies, nicotine-related regulations do not appear to follow a predictable progression based on evaluation timing alone.

Future evaluations of tobacco control policies would benefit from explicitly aligning outcome selection and evaluation timing with policy logic models (57) that articulate expected causal pathways and intermediate milestones. Such approaches can help distinguish between early system-level changes and downstream behavioral or health outcomes and improve the interpretability of observed policy effects across different evaluation lags. Greater use of theory-informed evaluation frameworks (58) may strengthen the comparability and policy relevance of future studies.

## Strengths and limitations

A key strength of this review is its focus on the temporal aspect of policy evaluation timing, which is often overlooked in policy evaluation studies. By synthesizing evidence across multiple policy domains, we highlight important differences in evaluation timing that can inform both research and practice. However, there are limitations to this review. First, we focused on studies from four HIC, potentially limiting generalizability to lower- and middle-income settings. We also acknowledge the exclusion of other HIC as limitation and an important direction for future research. Second, we did not include all local and state-level policies from these countries, which limit the effectiveness of the findings to the national level. Third, heterogeneity in study designs, outcomes, and definitions of “effectiveness” also constrains comparability across studies and the ability to conduct a meta-analysis of “effectiveness”. Fourth, our review did not synthesize findings on policy effects across diverse population groups as only three studies (15, 16, 20) conducted such evaluations. Future reviews to systematically examine policy effects across diverse population groups could provide important insights into policy effects on vulnerable and priority population. Lastly, publication bias and the exclusion of non-English studies may have further narrowed the evidence base. Nevertheless, future research can examine if there are differences in these countries relative to our study population.

## Conclusion

These findings reveal that PPELs vary by policy type. Smoke-free policies are evaluated earlier for behavioral outcomes after implementation than retail and nicotine-related policies. Evaluation lags are influenced by contextual factors such as jurisdictional variability, enforcement strategies, and the overall policy landscape. Nevertheless, our findings have implications for improving public health, it supports waiting through these lags to evaluate policy effectiveness, and where feasible, conduct repeated assessments which will lead to improved health outcomes.

## Data Availability

The original contributions presented in the study are included in the article/Supplementary material, further inquiries can be directed to the corresponding author.
