# Supplementary material for: Post-policy evaluation lags of tobacco and nicotine product policies: a systematic review from the United States, Canada, Australia, and the United Kingdom
Source: Front Public Health. 2026 Sep 29;14:1895110. doi: 10.3389/fpubh.2026.1895110 (PMC13658599; doi:10.3389/fpubh.2026.1895110)
Supplement: Supplementary file 1 [file Data_Sheet_1.doc]

**Post-Policy Evaluation Lags of Tobacco and Nicotine Product Policies: A Systematic Review from the USA, Canada, Australia, and the UK**

Supplemental Online Material

**Table 1.** **SEARCH STRATEGY**

| **Topic** | **Search words** |
| --- | --- |
| Abstract | Federal or National or Country or Nation |
| Title or Abstract or Keyword | Tobacco or smoking or nicotine or cigarettes or e-cigarettes or vape or vape pen or vap*or electronic nicotine delivery systems or ENDS or cigar or cigar*. |
| Title or Abstract or Keyword | Public policy or legislation or policy or Health policy or tobacco control policy or tobacco control act or tobacco control restrictions or restrictions or prohibitions or ban or statute or law or regulation or tobacco control |
| Abstract or Title | USA or US or United States of America or America or Canada or Australia or the United Kingdom or UK or England or Wales or Northern Ireland or Scotland |
| **Filters** | |
| Publication Years | 2010 to till date |
| Language | English |
| Document Type | Articles or original study articles published as full paper |
| Countries | U.S.A. or Canada or Australia or the United Kingdom |

**Web of Science**

**Results: 1324**

#1 AND #2 AND #3 AND #4

TI=("Tobacco" or "smoking" or "nicotine" or "cigarettes" or "e-cigarettes" or "vape" or "vape pen" or vap*or "electronic nicotine delivery systems" or "ENDS" or “cigar” or “cigar*****”) OR AB=("Tobacco" or "smoking" or "nicotine" or "cigarettes" or "e-cigarettes" or "vape" or "vape pen" or vap*or "electronic nicotine delivery systems" or "ENDS" or “cigar” or “cigar*****”) OR AK=("Tobacco" or "smoking" or "nicotine" or "cigarettes" or "e-cigarettes" or "vape" or "vape pen" or vap*or "electronic nicotine delivery systems" or "ENDS" or “cigar” or “cigar*****”)

TI=("Public policy" or "legislation" or "policy" or "Health policy" or "tobacco control policy" or "tobacco control act "or "tobacco control restrictions" or “restrictions” or “restriction” or “ prohibition” or “ban” or “statute” or “law” or “regulation” or “tobacco control”) OR AB=("Public policy" or "legislation" or "policy" or "Health policy" or "tobacco control policy" or "tobacco control act "or "tobacco control restrictions" ) OR AK= ("Public policy" or "legislation" or "policy" or "Health policy" or "tobacco control policy" or "tobacco control act "or "tobacco control restrictions" or “restriction” or “ prohibition” or “ban” or “statute” or “law” or “regulation” or “tobacco control”)

"USA" or "US" or "United States of America" or "America" or "Canada" or "Australia" or "the United Kingdom" or "UK" or "England" or "Wales" or "Northern Ireland" or "Scotland" (Title) or "USA" or "US" or "United States of America" or "America" or "Canada" or "Australia" or "the United Kingdom" or "UK" or "England" or "Wales" or "Northern Ireland" or "Scotland" (Abstract)

"Federal" or "National" or "Country" or "Nation" (Abstract)

Filters:

| Document Type | Article |
| --- | --- |
| NOT Document Type | Review Article |
| Languages | English |
| Countries/Regions | USA or England or Canada or Australia or Scotland |

**PubMed**

**Results: 432**

(("Tobacco"[Title/Abstract] OR "smoking"[Title/Abstract] OR "nicotine"[Title/Abstract] OR "cigarettes"[Title/Abstract] OR "e-cigarettes"[Title/Abstract] OR "vape"[Title/Abstract] OR "vape pen"[Title/Abstract] OR vap*or "electronic nicotine delivery systems"[Title/Abstract] OR "ENDS"[Title/Abstract] OR “cigar” [Title/Abstract] OR “cigar*”[Title/Abstract]) OR ("Tobacco"[Text Word] OR "smoking"[Text Word] OR "nicotine"[Text Word] OR "cigarettes"[Text Word] OR "e-cigarettes"[Text Word] OR "vape"[Text Word] OR "vape pen"[Text Word] OR vap*or "electronic nicotine delivery systems"[Text Word] OR "ENDS"[Text Word] OR “cigar” OR “cigar*”)) AND ("Public policy"[Title/Abstract] OR "legislation"[Title/Abstract] OR "policy"[Title/Abstract] OR "Health policy"[Title/Abstract] OR "tobacco control policy"[Title/Abstract] OR "tobacco control act "or "tobacco control restrictions"[Title/Abstract] OR “restriction”[Title/Abstract] OR “prohibition” [Title/Abstract] OR “ban” [Title/Abstract] OR “statute” [Title/Abstract] OR “law” [Title/Abstract] OR “regulation” [Title/Abstract] OR “tobacco control” [Title/Abstract] )) OR ("Public policy"[Text Word] OR "legislation"[Text Word] OR "policy"[Text Word] OR "Health policy"[Text Word] OR "tobacco control policy"[Text Word] OR "tobacco control act "or "tobacco control restrictions"[Text Word] OR “restriction” [Text Word] or “ prohibition” [Text Word] or “ban” [Text Word] or “statute” [Text Word] or “law” [Text Word] or “regulation” [Text Word] or “tobacco control”[Text Word])) AND ("USA"[Title/Abstract] OR "US"[Title/Abstract] OR "United States of America"[Title/Abstract] OR "America"[Title/Abstract] OR "Canada"[Title/Abstract] OR "Australia"[Title/Abstract] OR "the United Kingdom"[Title/Abstract] OR "UK"[Title/Abstract] OR "England"[Title/Abstract] OR "Wales"[Title/Abstract] OR "Northern Ireland"[Title/Abstract] OR "Scotland"[Title/Abstract] OR "USA"[Title/Abstract] OR "US"[Title/Abstract] OR "United States of America"[Title/Abstract] OR "America"[Title/Abstract] OR "Canada"[Title/Abstract] OR "Australia"[Title/Abstract] OR "the United Kingdom"[Title/Abstract] OR "UK"[Title/Abstract] OR "England"[Title/Abstract] OR "Wales"[Title/Abstract] OR "Northern Ireland"[Title/Abstract] OR "Scotland"[Title/Abstract])) AND ("Federal"[Title/Abstract] OR "National"[Title/Abstract] OR "Country"[Title/Abstract] OR "Nation"[Title/Abstract])) NOT ("Federal"[Title] OR "National"[Title] OR "Country"[Title] OR "Nation"[Title])

| Filters |  |
| --- | --- |
| Date | 2010-2025 |
| Article Type | Congress, Corrected and Republished Article, Duplicate Publication, Evaluation Study, Government Publication, Multicenter Study, Observational Study, Overall |
| Text Availability | Free full text, Full text. |

**Embase**

**Result: 1109**

('tobacco':ti,ab,kw OR 'smoking':ti,ab,kw OR 'nicotine':ti,ab,kw OR 'cigarettes':ti,ab,kw OR 'e-cigarettes':ti,ab,kw OR 'vape':ti,ab,kw OR 'vape pen':ti,ab,kw OR 'electronic nicotine delivery systems':ti,ab,kw OR 'ends':ti,ab,kw OR 'cigar':ti,ab,kw) AND ('public policy':ti,ab,kw OR 'legislation':ti,ab,kw OR 'policy':ti,ab,kw OR 'health policy':ti,ab,kw OR 'tobacco control policy':ti,ab,kw OR 'tobacco control act' OR 'tobacco control restrictions':ti,ab,kw OR ‘restriction’:ti,ab,kw OR ‘prohibition’:ti,ab,kw OR ‘ban’:ti,ab,kw OR ‘statute’:ti,ab,kw OR ‘law’:ti,ab,kw OR ‘regulation’:ti,ab,kw OR ‘tobacco control’:ti,ab,kw ) AND ('usa':ti,ab OR 'us':ti,ab OR 'united states of america':ti,ab OR 'america':ti,ab OR 'canada':ti,ab OR 'australia':ti,ab OR 'the united kingdom':ti,ab OR 'uk':ti,ab OR 'england':ti,ab OR 'wales':ti,ab OR 'northern ireland':ti,ab OR 'scotland':ti,ab) AND ('federal':ab OR 'national':ab OR 'country':ab OR 'nation':ab) AND 'article'/it AND [english]/lim AND ([embase]/lim OR [medline]/lim) AND [2010-2025]/py

**Google Scholar**

**RESULTS: 278**

[((((((Federal OR National OR Country OR Nation) AND (Tobacco OR smoking OR nicotine OR cigarettes OR e-cigarettes OR vape OR vape pen OR electronic nicotine delivery systems OR ENDS )) OR (Tobacco[Title/Abstract] OR smoking[Title/Abstract] OR nicotine[Title/Abstract] OR cigarettes[Title/Abstract] OR e-cigarettes[Title/Abstract] OR vape[Title/Abstract] OR vape pen[Title/Abstract] OR electronic nicotine delivery systems[Title/Abstract] OR ENDS[Title/Abstract])) AND (Public policy[Title/Abstract] OR legislation[Title/Abstract] OR policy[Title/Abstract] OR Health policy[Title/Abstract] OR tobacco control policy[Title/Abstract] OR tobacco control act[Title/Abstract] OR tobacco control restrictions[Title/Abstract])) AND (USA[Title/Abstract] OR US[Title/Abstract] OR United States of America[Title/Abstract] OR America[Title/Abstract] OR Canada[Title/Abstract] OR Australia[Title/Abstract] OR the United Kingdom[Title/Abstract] OR UK[Title/Abstract] OR England[Title/Abstract] OR Wales[Title/Abstract] OR Northern Ireland[Title/Abstract] OR Scotland[Title/Abstract])) AND ((fft[Filter]) AND (2010/1/1:2025[pdat]) AND (english[Filter])) AND ((fft[Filter]) AND (2010/1/1:2025[pdat]) AND (english[Filter])) Filters: Full text, English, from 2010/1/1 - 2025](https://scholar.google.com/scholar?q=((((((Federal+OR+National+OR+Country+OR+Nation)+AND+(Tobacco+OR+smoking+OR+nicotine+OR+cigarettes+OR+e-cigarettes+OR+vape+OR+vape+pen+OR+electronic+nicotine+delivery+systems+OR+ENDS+))+OR+(Tobacco%5BTitle/Abstract%5D+OR+smoking%5BTitle/Abstract%5D+OR+nicotine%5BTitle/Abstract%5D+OR+cigarettes%5BTitle/Abstract%5D+OR+e-cigarettes%5BTitle/Abstract%5D+OR+vape%5BTitle/Abstract%5D+OR+vape+pen%5BTitle/Abstract%5D+OR+electronic+nicotine+delivery+systems%5BTitle/Abstract%5D+OR+ENDS%5BTitle/Abstract%5D))+AND+(Time+OR+timing+OR+timed+OR+period))+AND+(Public+policy%5BTitle/Abstract%5D+OR+legislation%5BTitle/Abstract%5D+OR+policy%5BTitle/Abstract%5D+OR+Health+policy%5BTitle/Abstract%5D+OR+tobacco+control+policy%5BTitle/Abstract%5D+OR+tobacco+control+act%5BTitle/Abstract%5D+OR+tobacco+control+restrictions%5BTitle/Abstract%5D))+AND+(USA%5BTitle/Abstract%5D+OR+US%5BTitle/Abstract%5D+OR+United+States+of+America%5BTitle/Abstract%5D+OR+America%5BTitle/Abstract%5D+OR+Canada%5BTitle/Abstract%5D+OR+Australia%5BTitle/Abstract%5D+OR+the+United+Kingdom%5BTitle/Abstract%5D+OR+UK%5BTitle/Abstract%5D+OR+England%5BTitle/Abstract%5D+OR+Wales%5BTitle/Abstract%5D+OR+Northern+Ireland%5BTitle/Abstract%5D+OR+Scotland%5BTitle/Abstract%5D))+AND+((fft%5BFilter%5D)+AND+(2010/1/1:2025%5Bpdat%5D)+AND+(english%5BFilter%5D))+AND+((fft%5BFilter%5D)+AND+(2010/1/1:2025%5Bpdat%5D)+AND+(english%5BFilter%5D))+Filters:+Full+text,+English,+from+2010/1/1+-+2025&hl=en&as_sdt=0,15&scilib=1&scioq=((((((Federal+OR+National+OR+Country+OR+Nation)+AND+(Tobacco+OR+smoking+OR+nicotine+OR+cigarettes+OR+e-cigarettes+OR+vape+OR+vape+pen+OR+electronic+nicotine+delivery+systems+OR+ENDS+))+OR+(Tobacco%5BTitle/Abstract%5D+OR+smoking%5BTitle/Abstract%5D+OR+nicotine%5BTitle/Abstract%5D+OR+cigarettes%5BTitle/Abstract%5D+OR+e-cigarettes%5BTitle/Abstract%5D+OR+vape%5BTitle/Abstract%5D+OR+vape+pen%5BTitle/Abstract%5D+OR+electronic+nicotine+delivery+systems%5BTitle/Abstract%5D+OR+ENDS%5BTitle/Abstract%5D))+AND+(Time+OR+timing+OR+timed+OR+period))+AND+(Public+policy%5BTitle/Abstract%5D+OR+legislation%5BTitle/Abstract%5D+OR+policy%5BTitle/Abstract%5D+OR+Health+policy%5BTitle/Abstract%5D+OR+tobacco+control+policy%5BTitle/Abstract%5D+OR+tobacco+control+act%5BTitle/Abstract%5D+OR+tobacco+control+restrictions%5BTitle/Abstract%5D))+AND+(USA%5BTitle/Abstract%5D+OR+US%5BTitle/Abstract%5D+OR+United+States+of+America%5BTitle/Abstract%5D+OR+America%5BTitle/Abstract%5D+OR+Canada%5BTitle/Abstract%5D+OR+Australia%5BTitle/Abstract%5D+OR+the+United+Kingdom%5BTitle/Abstract%5D+OR+UK%5BTitle/Abstract%5D+OR+England%5BTitle/Abstract%5D+OR+Wales%5BTitle/Abstract%5D+OR+Northern+Ireland%5BTitle/Abstract%5D+OR+Scotland%5BTitle/Abstract%5D))+AND+((fft%5BFilter%5D)+AND+(2010/1/1:2025%5Bpdat%5D)+AND+(english%5BFilter%5D))+AND+((fft%5BFilter%5D)+AND+(2010/1/1:2025%5Bpdat%5D)+AND+(english%5BFilter%5D))+Filters:+Full+text,+English,+from+2010/1/1+-+2025)

**SCOPUS (First Search Results)**

**RESULTS = 493**

( ALL ( ( federal OR national ) ) AND TITLE-ABS-KEY ( tobacco OR smoking OR nicotine OR cigarettes ) AND ALL ( time OR timing OR timed ) AND TITLE-ABS-KEY ( Public policy OR legislation OR policy ) AND ALL ( evaluation OR assessment OR examination ) AND ALL ( U.S.A OR Australia OR Canada OR United Kingdom ) ) AND PUBYEAR > 2009 AND PUBYEAR < 2024 AND PUBYEAR > 2009 AND PUBYEAR < 2024 AND ( LIMIT-TO ( AFFILCOUNTRY , "United Kingdom" ) OR LIMIT-TO ( AFFILCOUNTRY , "United States" ) OR LIMIT-TO ( AFFILCOUNTRY , "Australia" ) OR LIMIT-TO ( AFFILCOUNTRY , "Canada" ) ) AND ( LIMIT-TO ( SUBJAREA , "MEDI" ) OR LIMIT-TO ( SUBJAREA , "SOCI" ) OR LIMIT-TO ( SUBJAREA , "NURS" ) OR LIMIT-TO ( SUBJAREA , "MULT" ) OR LIMIT-TO ( SUBJAREA , "ENVI" ) OR LIMIT-TO ( SUBJAREA , "PSYC" ) OR LIMIT-TO ( SUBJAREA , "HEAL" ) OR LIMIT-TO ( SUBJAREA , "DENT" ) ) AND ( LIMIT-TO ( DOCTYPE , "ar" ) OR LIMIT-TO ( DOCTYPE , "re" ) ) AND ( LIMIT-TO ( LANGUAGE , "English" ) )

**Grey Literature**

The following government websites from Australia, the United States, the United Kingdom (including England, Northern Ireland, Scotland, and Wales), and Canada were searched to retrieve relevant studies.

- Catalog of U.S. Government Publications (CGP)
- Government of Canada Publications website
- Scottish Government Statistics and Research
- Official Statistics and Research on Wales.
- Government of United Kingdom Research and statistics.
- Northern Ireland Statistics and Research Agency (NISRA).
- Analysis and Policy Observatory (APO) for Australia.

**Table 2: Policy Related Characteristics of Final Sample (N= 21)**

| **Study** | **Country** | **Policy Name** | **Implementation Date** | **Evaluation Timepoints** | **Post Policy Evaluation lag** | **Policy Implementation date** | | |
| --- | --- | --- | --- | --- | --- | --- | --- | --- |
|  |  |  |  |  |  | **2010 or greater** | | **Clearly mentioned in the Study** |
| **Smoke-free policies (n =04)** | | | | | | | | |
| Elton-Marshall 2015^1^ | Canada | Ban smoking in cars.  Smoke-free across most provinces.  Our review included only provinces with policy implementation date of 2010 and onwards | 2010 (Manitoba, Saskatchewan)  2011 (Newfoundland/  Labrador) | 2010 (Manitoba)  2010, 2012 (Saskatchewan)  2012 (Newfoundland/  Labrador) | Within six months to one year (Manitoba, Saskatchewan)  One to two years (Saskatchewan, Newfoundland/  Labrador) | Yes | Yes | |
| Thorpe 2020^2^ | USA | Federal smoke-free housing policy  Smoke-free housing | July, 2018 | 2018-2019 | Within six months to one year | Yes | Yes | |
| Tweed 2021^3^ | Scotland | Smoke-free prison policy | Nov. 30, 2018 | 2014 - 2019 | Within six months to one year | Yes | Yes | |
| Hunt 2022^4^ | Scotland | Comprehensive smoke-free prison policy | 2018 | 2016-2020 | One to two years | Yes | Yes | |
| **Retail-related policies** | | | | | | | | |
| **Subcategory- Cigarette Flavor ban (n =04)** | | | | | | | | |
| Carpenter 2021^5^ | Canada | Menthol Flavor Ban (cigarettes)  Flavor ban across most provinces | 2015 (Nova Scotia, Alberta)  2016 (New Brunswick, Quebec)  2017 (Ontario, Prince Edward Island, Newfoundland/Labrador)  2017 (Federal Ban) | 2010-2017 (for youth)  2003-2017 (for adults) - | One to two years  (Provinces are treated as treatment and control groups based on the ban implementation) | Yes | Yes | |
| Chung-Hall 2022^6^ | Canada | Menthol Flavor Ban (cigarettes)  Flavor ban across most provinces | 2017* | 2016-  July 2018 | One to two years | Yes | Yes | |
| Chung-Hall 2024^7^ | Canada | Menthol Flavor Ban (cigarettes)  Flavor ban across most provinces | 2017* | 2016-  July 2018 | One to two years | Yes | Yes | |
| East 2022^8^ | Canada, England, USA | Menthol Flavor ban (cigarettes)  Flavor ban on Federal level | Canada = Oct 2017  England = May 2020  USA= none | 2018, 2019, February and August 2020 | Within six months to one year | Yes | Yes | |
| **Subcategory – Packaging/point of sale (n = 09)** | | | | | | | | |
| Opazo Breton 2023^9^ | United Kingdom | Standardized Packaging Tobacco Products Regulation (SPoT) | May 2016 | May 2016 and May 2017 | Within six months to one year | Yes | Yes | |
| Buss 2024^10^ | United Kingdom | Standardized Packaging Tobacco Products Regulation (SPoT): for cigarettes and hand rolling tobacco | May 2016 | November 2007 - January 2020 | Over two years | Yes | Yes | |
| Review of Tobacco Legislations (Grey Literature)^11^ | United Kingdom | Standardized Packaging Tobacco Products Regulation (SPoT): for cigarettes and hand rolling tobacco | May 2016 | 2015 - 2018 | One to two years | Yes | Yes | |
| Review of Tobacco Legislations (Grey Literature)^12^ | United Kingdom | Tobacco and Related Products Regulation (TRPR) | May 2016 | 2015 - 2018 | One to two years | Yes | Yes | |
| Durkin 2015^13^ | Australia | Tobacco Plain Packaging act | Late 2012 | 2012 - 2014 | One to two years | Yes | Yes | |
| Underwood 2020^14^ | Australia | Tobacco Plain Packaging act | December 2012 | 2002 - 2017 | Over two years | Yes | Yes | |
| Thrasher 2015^15^ | Canada | Cigarette package inserts  To supplement Pictorial Health warnings | June 2012 | September 2012- January 2014 | One to two years | Yes | Yes | |
| Kuipers 2017^16^ | England | Partial tobacco point of sale display ban  Banning all tobacco displays from larger shops. | April 6, 2012 | January 2009 -February 2015 | Over two years | Yes | Yes | |
| Agaku 2022^17^ | USA | Tobacco 21 in California, Hawaii, New Jersey, Oregon, Maine, and Massachusetts | 2016-2018 | 2019 | Over two years (California, Hawaii)  One to two years (Oregon, Maine, New Jersey and Massachusetts) | Yes | Yes | |
| **Subcategory – Nicotine-related (n = 03)** | | | | | | | | |
| Mendelsohn 2025^18^ | Australia  New Zealand | Australia classified nicotine e-liquid as a prescription-only ‘unapproved’ medicine since 2011  Nicotine e-liquid has been legally available in New Zealand as an unregulated adult consumer product since 2010. | Australia = 2011  New Zealand = 2010 | 2016 - 2023 | Over two years | Yes | Yes | |
| Moore 2020^19^ | England, Wales and Scotland | Tobacco Products Directive (TPD) regulations | May 2016  to May 2017 | 2015 -2017 | One to two years | Yes | Yes | |
| Hammond 2020^20^ | Canada | Tobacco and Vaping Product Act (TPVA) | May 2018 | 2017, 2018, 2019 | Within six months to one year (2018)  One to two years (2019) | Yes | Yes | |
| **Subcategory – Tax Policy (n = 01)** | | | | | | | | |
| Havard 2018^21^ | Australia | 25% tobacco excise and customs duty increase | April 2010 | July 2010 -Dec. 2011 | One to two years | Yes | Yes | |

**Researchers considered the date for which last province implemented ban*

**Table 3. Risk of Bias Assessment Tool**

| **No.** | **Checklist** |
| --- | --- |
|  | **Internal Validity** |
| 01 | Criteria of Inclusion are clearly defined |
| 02 | Subjects and settings described in detail |
| 03 | Confounding factors identified |
| 04 | Use of objective, standard criteria to measure condition |
|  | **Statistical Validity** |
| 05 | Exposure measured in a valid & reliable way |
| 06 | Strategies to deal with confounding factors |
| 07 | Outcome measured in a valid and reliable way |
| 08 | Appropriate statistical analysis was used |

**Table 4: Quality Assessment of Full Sample (N =21)**

| **Study** | **Risk of Bias: Internal Validity** | **Risk of Bias: Statistical Conclusion** | **Overall Risk of Bias** |
| --- | --- | --- | --- |
| Elton-Marshall 2015 | Low | Low | Low |
| Thorpe 2020 | Low | Low | Low |
| Tweed 2021 | Low | Low | Low |
| Hunt 2022 | Low | Low | Low |
| Carpenter 2021 | Low | Low | Low |
| Chung-Hall 2022 | Low | Low | Low |
| Chung-Hall 2024 | Low | Low | Low |
| East 2022 | Low | Low | Low |
| Opazo Breton 2023 | Low | Low | Low |
| Buss 2024 | Low | Low | Low |
| Durkin 2015 | Low | Low | Low |
| Underwood 2020 | Low | Low | Low |
| Thrasher 2015 | Low | Low | Low |
| Kuipers 2017 | Low | Low | Low |
| Moore 2020 | Low | Low | Low |
| Hammond 2020 | Low | Low | Low |
| Havard 2018 | Low | Low | Low |
| Agaku 2022 | Low | Low | Low |
| Mendelsohn 2025 | Unclear: Absence of adjustment for key confounders, illicit market access, and media influence. The difference in survey instruments and age ranges between Australia and New Zealand leads to inconsistencies. | Low | Low |
| Gray literature  (The Standardised Packaging of Tobacco Products Regulations 2015) | Unclear: The document is a combination of information collected from public consultations, peer reviewed evidence and other publicly available data | Unclear: It is a review report generated based on existing evidence with no sound statistical foundation to control confounders while evaluating policy impact. | Unclear |
| Gray literature  (The Tobacco and Related Products Regulations 2016) | Unclear: The document is a combination of information collected from public consultations, peer reviewed evidence and other publicly available data. | Unclear: It is a review report generated based on existing evidence with no sound statistical foundation to control confounders while evaluating policy impact. | Unclear |

# **PRISMA 2020 Main Checklist**

| **Topic** | **No.** | **Item** | **Location where item is reported** |
| --- | --- | --- | --- |
| **TITLE** |  |  |  |
| **Title** | 1 | Identify the report as a systematic review. | Title |
| **ABSTRACT** |  |  |  |
| **Abstract** | 2 | See the PRISMA 2020 for Abstracts checklist | Supplementary document, Abstract checklist |
| **INTRODUCTION** |  |  |  |
| **Rationale** | 3 | Describe the rationale for the review in the context of existing knowledge. | Introduction section |
| **Objectives** | 4 | Provide an explicit statement of the objective(s) or question(s) the review addresses. | Introduction section |
| **METHODS** |  |  |  |
| **Eligibility criteria** | 5 | Specify the inclusion and exclusion criteria for the review and how studies were grouped for the syntheses. | Methods section |
| **Information sources** | 6 | Specify all databases, registers, websites, organisations, reference lists and other sources searched or consulted to identify studies. Specify the date when each source was last searched or consulted. | Methods section |
| **Search strategy** | 7 | Present the full search strategies for all databases, registers and websites, including any filters and limits used. | Supplementary material, Table 1 |
| **Selection process** | 8 | Specify the methods used to decide whether a study met the inclusion criteria of the review, including how many reviewers screened each record and each report retrieved, whether they worked independently, and if applicable, details of automation tools used in the process. | Methods section |
| **Data collection process** | 9 | Specify the methods used to collect data from reports, including how many reviewers collected data from each report, whether they worked independently, any processes for obtaining or confirming data from study investigators, and if applicable, details of automation tools used in the process. | Methods section |
| **Data items** | 10a | List and define all outcomes for which data were sought. Specify whether all results that were compatible with each outcome domain in each study were sought (e.g. for all measures, time points, analyses), and if not, the methods used to decide which results to collect. | Measures section |
|  | 10b | List and define all other variables for which data were sought (e.g. participant and intervention characteristics, funding sources). Describe any assumptions made about any missing or unclear information. | Measures section |
| **Study risk of bias assessment** | 11 | Specify the methods used to assess risk of bias in the included studies, including details of the tool(s) used, how many reviewers assessed each study and whether they worked independently, and if applicable, details of automation tools used in the process. | Quality assessment section |
| **Effect measures** | 12 | Specify for each outcome the effect measure(s) (e.g. risk ratio, mean difference) used in the synthesis or presentation of results. | Methods section |
| **Synthesis methods** | 13a | Describe the processes used to decide which studies were eligible for each synthesis (e.g. tabulating the study intervention characteristics and comparing against the planned groups for each synthesis (item 5)). | Methods section |
|  | 13b | Describe any methods required to prepare the data for presentation or synthesis, such as handling of missing summary statistics, or data conversions. | Not applicable |
|  | 13c | Describe any methods used to tabulate or visually display results of individual studies and syntheses. | Figure 1-2, Table 1-3 |
|  | 13d | Describe any methods used to synthesize results and provide a rationale for the choice(s). If meta-analysis was performed, describe the model(s), method(s) to identify the presence and extent of statistical heterogeneity, and software package(s) used. | Methods section |
|  | 13e | Describe any methods used to explore possible causes of heterogeneity among study results (e.g. subgroup analysis, meta-regression). | Not applicable |
|  | 13f | Describe any sensitivity analyses conducted to assess robustness of the synthesized results. | Not applicable |
| **Reporting bias assessment** | 14 | Describe any methods used to assess risk of bias due to missing results in a synthesis (arising from reporting biases). | Not applicable |
| **Certainty assessment** | 15 | Describe any methods used to assess certainty (or confidence) in the body of evidence for an outcome. | Not applicable |
| **RESULTS** |  |  |  |
| **Study selection** | 16a | Describe the results of the search and selection process, from the number of records identified in the search to the number of studies included in the review, ideally using a flow diagram. | Figure 1 |
|  | 16b | Cite studies that might appear to meet the inclusion criteria, but which were excluded, and explain why they were excluded. | Figure 1 |
| **Study characteristics** | 17 | Cite each included study and present its characteristics. | Table 3 and characteristics of the studies subsection of Methods section |
| **Risk of bias in studies** | 18 | Present assessments of risk of bias for each included study. | Table 2 |
| **Results of individual studies** | 19 | For all outcomes, present, for each study: (a) summary statistics for each group (where appropriate) and (b) an effect estimate and its precision (e.g. confidence/credible interval), ideally using structured tables or plots. | Table 1 |
| **Results of syntheses** | 20a | For each synthesis, briefly summarise the characteristics and risk of bias among contributing studies. | characteristics of studies and Quality assessment sub sections within Methods section |
|  | 20b | Present results of all statistical syntheses conducted. If meta-analysis was done, present for each the summary estimate and its precision (e.g. confidence/credible interval) and measures of statistical heterogeneity. If comparing groups, describe the direction of the effect. | Findings section |
|  | 20c | Present results of all investigations of possible causes of heterogeneity among study results. | Table 2 and Supplementary Table 2 & 4 |
|  | 20d | Present results of all sensitivity analyses conducted to assess the robustness of the synthesized results. | Not applicable |
| **Reporting biases** | 21 | Present assessments of risk of bias due to missing results (arising from reporting biases) for each synthesis assessed. | Supplementary Table 4 |
| **Certainty of evidence** | 22 | Present assessments of certainty (or confidence) in the body of evidence for each outcome assessed. | - |
| **DISCUSSION** |  |  |  |
| **Discussion** | 23a | Provide a general interpretation of the results in the context of other evidence. | Discussion section |
|  | 23b | Discuss any limitations of the evidence included in the review. | Discussion section |
|  | 23c | Discuss any limitations of the review processes used. | Discussion section |
|  | 23d | Discuss implications of the results for practice, policy, and future research. | Discussion section |
| **OTHER INFORMATION** |  |  |  |
| **Registration and protocol** | 24a | Provide registration information for the review, including register name and registration number, or state that the review was not registered. | Methods section |
|  | 24b | Indicate where the review protocol can be accessed, or state that a protocol was not prepared. | Methods section |
|  | 24c | Describe and explain any amendments to information provided at registration or in the protocol. | Not applicable |
| **Support** | 25 | Describe sources of financial or non-financial support for the review, and the role of the funders or sponsors in the review. | Declaration section |
| **Competing interests** | 26 | Declare any competing interests of review authors. | Declaration section |
| **Availability of data, code and other materials** | 27 | Report which of the following are publicly available and where they can be found: template data collection forms; data extracted from included studies; data used for all analyses; analytic code; any other materials used in the review. | Declaration section |

# **PRIMSA Abstract Checklist**

| **Topic** | **No.** | **Item** | **Reported?** |
| --- | --- | --- | --- |
| **TITLE** |  |  |  |
| **Title** | 1 | Identify the report as a systematic review. | Yes |
| **BACKGROUND** |  |  |  |
| **Objectives** | 2 | Provide an explicit statement of the main objective(s) or question(s) the review addresses. | Yes |
| **METHODS** |  |  |  |
| **Eligibility criteria** | 3 | Specify the inclusion and exclusion criteria for the review. | Yes |
| **Information sources** | 4 | Specify the information sources (e.g. databases, registers) used to identify studies and the date when each was last searched. | Yes |
| **Risk of bias** | 5 | Specify the methods used to assess risk of bias in the included studies. | Yes |
| **Synthesis of results** | 6 | Specify the methods used to present and synthesize results. | Yes |
| **RESULTS** |  |  |  |
| **Included studies** | 7 | Give the total number of included studies and participants and summarise relevant characteristics of studies. | Yes |
| **Synthesis of results** | 8 | Present results for main outcomes, preferably indicating the number of included studies and participants for each. If meta-analysis was done, report the summary estimate and confidence/credible interval. If comparing groups, indicate the direction of the effect (i.e. which group is favoured). | Yes |
| **DISCUSSION** |  |  |  |
| **Limitations of evidence** | 9 | Provide a brief summary of the limitations of the evidence included in the review (e.g. study risk of bias, inconsistency and imprecision). | Yes |
| **Interpretation** | 10 | Provide a general interpretation of the results and important implications. | Yes |
| **OTHER** |  |  |  |
| **Funding** | 11 | Specify the primary source of funding for the review. | No |
| **Registration** | 12 | Provide the register name and registration number. | Yes |

References of Studies included in the Review:

1.	Elton-Marshall T, Leatherdale ST, Driezen P, Azagba S, Burkhalter R. Do provincial policies banning smoking in cars when children are present impact youth exposure to secondhand smoke in cars? *Prev Med*. 2015;78:59-64. doi:10.1016/j.ypmed.2015.07.007

2.	Thorpe LE, Anastasiou E, Wyka K, et al. Evaluation of Secondhand Smoke Exposure in New York City Public Housing After Implementation of the 2018 Federal Smoke-Free Housing Policy. *JAMA Netw Open*. 2020;3(11):e2024385. doi:10.1001/jamanetworkopen.2020.24385

3.	Tweed EJ, Mackay DF, Boyd KA, et al. Evaluation of a national smoke-free prisons policy using medication dispensing: an interrupted time-series analysis. *Lancet Public Health*. 2021;6(11):e795-e804. doi:10.1016/S2468-2667(21)00163-8

4.	Hunt K, Brown A, Eadie D, et al. Process and impact of implementing a smoke-free policy in prisons in Scotland: TIPs mixed-methods study. 2022;10. doi:10.3310/WGLF1204

5.	Carpenter C, Nguyen H. Intended and Unintended Effects of Banning Menthol Cigarettes. *J LAW Econ*. 2021;64(3):629-650. doi:10.1086/713978

6.	Chung-Hall J, Fong GT, Meng G, et al. Evaluating the impact of menthol cigarette bans on cessation and smoking behaviours in Canada: longitudinal findings from the Canadian arm of the 2016–2018 ITC Four Country Smoking and Vaping Surveys. *Tob Control*. 2022;31(4):556. doi:10.1136/tobaccocontrol-2020-056259

7.	Chung-Hall J, Fong GT, Meng G, Craig LV. Illicit cigarette purchasing after implementation of menthol cigarette bans in Canada: findings from the 2016-2018 ITC Four Country Smoking and Vaping Surveys. *Tob Control*. 2024;33(e1):e128-e131. doi:10.1136/tc-2022-057697

8.	East KA, Reid JL, Burkhalter R, et al. Evaluating the Outcomes of the Menthol Cigarette Ban in England by Comparing Menthol Cigarette Smoking Among Youth in England, Canada, and the US, 2018-2020. *JAMA Netw Open*. 2022;5(5):e2210029-e2210029. doi:10.1001/jamanetworkopen.2022.10029

9.	Opazo Breton M, Britton J, Brown J, Beard E, Bogdanovica I. Was the implementation of standardised tobacco packaging legislation in England associated with changes in smoking prevalence? A segmented regression analysis between 2006 and 2019. *Tob Control*. 2023;32(2):195-204. doi:10.1136/tobaccocontrol-2021-056694

10.	Buss VH, Kock L, Beard E, Shahab L, Brown J, Jackson S. Impact of Standardised Packaging of Tobacco Products Regulations on cigarette consumption and youth smoking in England: interrupted time-series analysis. *Tob Control*. 2024;((Buss V.H., v.buss@ucl.ac.uk; Kock L.; Shahab L.; Brown J.; Jackson S.) Department of Behavioural Science and Health, University College London, London, United Kingdom(Buss V.H., buss@ucl.ac.uk; Beard E.; Shahab L.; Brown J.; Jackson S.) SPECTRUM Resear). doi:10.1136/tc-2023-058560

11.	*Post-Implementation Review of Tobacco Legislation: The Standardised Packaging of Tobacco Products Regulations 2015*. Dandy Booksellers Ltd; 2022. https://assets.publishing.service.gov.uk/media/623d86db8fa8f5277500d945/the-standardised-packaging-of-tobacco-products-regulations-2015-post-implementation-review-web-accessible.pdf

12.	*Post-Implementation Review of Tobacco Legislation: The Tobacco and Related Products Regulations 2016*. Department of Health & Social Care.; 2022. https://assets.publishing.service.gov.uk/media/623ca9dd8fa8f52773d769e8/the-tobacco-and-related-products-regulations-2016-post-implementation-review-web-accessible.pdf

13.	Durkin S, Brennan E, Coomber K, Zacher M, Scollo M, Wakefield M. Short-term changes in quitting-related cognitions and behaviours after the implementation of plain packaging with larger health warnings: findings from a national cohort study with Australian adult smokers. *Tob Control*. 2015;24(Suppl 2):ii26-ii32. doi:10.1136/tobaccocontrol-2014-052058

14.	Underwood D, Sun S, Welters RAMHM. The effectiveness of plain packaging in discouraging tobacco consumption in Australia. *Nat Hum Behav*. 2020;4(12):1273-1284. doi:10.1038/s41562-020-00940-6

15.	Thrasher J, Osman A, Abad-Vivero E, et al. The Use of Cigarette Package Inserts to Supplement Pictorial Health Warnings: An Evaluation of the Canadian Policy. *NICOTINE Tob Res*. 2015;17(7):870-875. doi:10.1093/ntr/ntu246

16.	Kuipers MA, Beard E, Hitchman SC, et al. Impact on smoking of England’s 2012 partial tobacco point of sale display ban: a repeated cross-sectional national study. *Tob Control*. 2017;26(2):141-148. doi:10.1136/tobaccocontrol-2015-052724

17.	Agaku I. A rapid evaluation of the US Federal Tobacco 21 (T21) Law and lessons from statewide T21 policies: Findings from population-level surveys. *Tob Prev Cessat*. 2022;8(Supplement). doi:10.18332/tpc/150914

18.	Mendelsohn C, Beaglehole R, Borland R, et al. Do the differing vaping and smoking trends in Australia and New Zealand reflect different regulatory policies? *ADDICTION*. Published online 2025. doi:10.1111/add.70006

19.	Moore G, Brown R, Page N, et al. Young people’s use of e-cigarettes in Wales, England and Scotland before and after introduction of EU Tobacco Products Directive regulations: a mixed-method natural experimental evaluation. *Int J Drug Policy*. 2020;85:102795. doi:10.1016/j.drugpo.2020.102795

20.	Hammond D, Reid JL, Burkhalter R, Rynard VL. E-cigarette Marketing Regulations and Youth Vaping: Cross-Sectional Surveys, 2017-2019. *Pediatrics*. 2020;146(1). doi:10.1542/peds.2019-4020

21.	Havard A, Tran DT, Kemp-Casey A, Einarsdóttir K, Preen DB, Jorm LR. Tobacco policy reform and population-wide antismoking activities in Australia: the impact on smoking during pregnancy. *Tob Control*. 2018;27(5):552-559. doi:10.1136/tobaccocontrol-2017-053715
